# Simultaneous Estimation of Vehicle Roll and Sideslip Angles through a Deep Learning Approach

**DOI:** 10.3390/s20133679

**Published:** 2020-06-30

**Authors:** Lisardo Prieto González, Susana Sanz Sánchez, Javier Garcia-Guzman, María Jesús L. Boada, Beatriz L. Boada

**Affiliations:** 1 Computer Science Department, Institute for Automotive Vehicle Safety (ISVA), Universidad Carlos III de Madrid, Avda. de la Universidad 30, 28911 Leganés, Madrid, Spain; jgarciag@inf.uc3m.es; 2 Mechanical Engineering Department, Institute for Automotive Vehicle Safety (ISVA), Universidad Carlos III de Madrid, Avda. de la Universidad 30, 28911 Leganés, Madrid, Spain; ssanz@ing.uc3m.es (S.S.S.); mjboada@ing.uc3m.es (M.J.L.B.); bboada@ing.uc3m.es (B.L.B.)

**Keywords:** sensor fusion, deep Learning based estimator, vehicle dynamics, roll angle, sideslip angle

## Abstract

Presently, autonomous vehicles are on the rise and are expected to be on the roads in the coming years. In this sense, it becomes necessary to have adequate knowledge about its states to design controllers capable of providing adequate performance in all driving scenarios. Sideslip and roll angles are critical parameters in vehicular lateral stability. The later has a high impact on vehicles with an elevated center of gravity, such as trucks, buses, and industrial vehicles, among others, as they are prone to rollover. Due to the high cost of the current sensors used to measure these angles directly, much of the research is focused on estimating them. One of the drawbacks is that vehicles are strong non-linear systems that require specific methods able to tackle this feature. The evolution in Artificial Intelligence models, such as the complex Artificial Neural Network architectures that compose the Deep Learning paradigm, has shown to provide excellent performance for complex and non-linear control problems. In this paper, the authors propose an inexpensive but powerful model based on Deep Learning to estimate the roll and sideslip angles simultaneously in mass production vehicles. The model uses input signals which can be obtained directly from onboard vehicle sensors such as the longitudinal and lateral accelerations, steering angle and roll and yaw rates. The model was trained using hundreds of thousands of data provided by Trucksim^®^ and validated using data captured from real driving maneuvers using a calibrated ground truth device such as VBOX3i dual-antenna GPS from Racelogic^®^. The use of both Trucksim^®^ software and the VBOX measuring equipment is recognized and widely used in the automotive sector, providing robust data for the research shown in this article.

## 1. Introduction

Road vehicles are the most prevalent transportation system. Current estimations set the mortality due to traffic accidents and collisions in around 1.35 million people per year [1]. Over the last several decades, vehicles have been equipped with active systems such as ABS (Anti-Blocking System), ESC (Electronic Stability Controllers), and active suspensions, which improve their comfort, efficiency, and safety. These systems gain greater interest in autonomous vehicles. The primary cause of accidents is related to loss of lateral stability control [2]. In this regard, sideslip and roll angles are critical parameters in vehicular lateral stability. It has become necessary to know about them to design controllers capable of providing adequate performance in all driving scenarios. Specifically, the roll angle has a high impact on vehicles with an elevated center of gravity, such as trucks, buses, and industrial vehicles, among others, as they are prone to rollover. Presently, there exist devices that allow the direct measurement of both angles, such as a dual antenna GPS (Global Positioning System) or a Kistler S-Motion device [3]. This type of advanced GPS equipment cannot be included in mass production vehicles due to high-cost issues [4]. Because of that, many of the researchers focus on estimating these angles based on available measurements from on-board embedded sensors but independently [5,6]. On the other hand, in works such as [7], both roll and sideslip angles are estimated by using additional external sensors that need to be integrated into the vehicle instead of using the own on-board systems. In general, these approaches are based on vehicle model-based estimation and data-driven-based estimation [8]. The former group includes approaches such as Kalman-filter-based method [9,10,11], nonlinear-observer-based method [11,12,13], and robust observers [14,15,16]. The latter includes Neural Networks, and ANFIS, among others [4,17,18], whose main advantage is that they do not depend on the reference vehicle models. Additionally, vehicles are strong non-linear systems that require specific methods able to tackle this feature. Presently, Machine Learning technologies are gaining attention in the field of improved vehicle driving, providing a diverse application set, ranging from computer vision for the identification of static or dynamic obstacles or the detection of fatigue situations in drivers to vehicle trajectory prediction [19,20,21,22]. Deep learning can be defined as a set of machine learning algorithms that implement large neural networks, including many hidden layers (also referred to as Deep Neural Networks—DNNs) for feature generation, learning, classification, and prediction [23]. There are several model architectures and transfer learning techniques that can be applied to solve different problems in the automotive domain. Nevertheless, more research is needed:

(a) to design distributed deep learning systems to improve training times for more complex networks and massive data sets;
(b) to determine how to apply deep learning in other areas of automobile control such as lateral stability [24]; and
(c) it is necessary to assess that the fusion of data coming from low-cost devices and estimations provided by deep machine learning algorithms can fulfill the reliability and appropriateness requirements for using these technologies to improve overall vehicular safety.

The novelty of this work consists of the design and implementation of an efficient and precise Deep Neural Network to simultaneously estimate roll and sideslip angles by using only information provisioned by on-board sensors such as the IMU (Inertial Measurement Unit) and the steering angle sensor. The designed DNN can tackle strong non-linear vehicle behavior. Besides, the proposed DNN does not use previous information from the sensors. Just the one provided at that time, so there are no stability problems associated with an accumulated error.

This article is organized as follows. Section 2 presents the steps taken to define the Deep Learning model for this research work, and the designed Deep Neural Network that solves the problem. Section 3 shows the results as far as predicted precision is concerned. Finally, a discussion related to the results, a set of conclusions, and the next steps to be taken by our research team are introduced in Section 4.

## 2. Methodology

This section describes the experimental approach adopted to achieve the goals stated for this research work. Showing up next is an overview of the different steps and required aspects before defining a proper Deep Neural Network. Then, different subsections detail each of the main aspects considered in the model creation.

**Figure 1:**
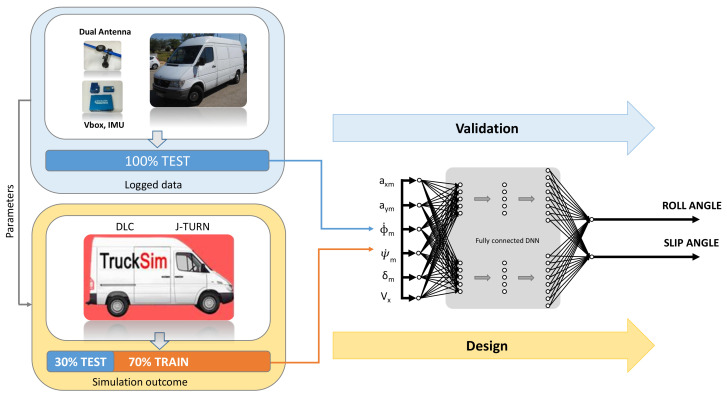
Training and validation scheme.

The main components that define our solutions are presented in Figure 1. They can be summarized as:

- Data set with repeatable simulated maneuvers with a complex vehicular model (van).
- Data set with information logged from real driving scenarios.
- Deep Learning predictor model, tested against the first data set.
- Validation of the Deep Learning Network using the second data set.

### 2.1. General Scheme

#### 2.1.1. Deep Learning Model to Accurately Predict Roll and Sideslip Angles

Presently, there exist a wide variety of deep neural networks (DNNs) and related architectures. For instance, recurrent neural networks (RNNs) [25] can be used in tasks as natural language processing (NLP), connected handwriting, speech recognition, or the generation of new sentences and document summaries [26]. Alternatively, long short-term memory (LSTM), initially designed to model temporal sequences considering their respective long-range dependencies with higher accuracy than conventional RNNs. LSTMs allow solving problems such as the creation of outstanding acoustic models for complex languages, and the tagging of parts of the speech with high precision [27,28,29,30], among others. Given the nature of the problem, a multilayer perceptron (MLP) has been used in this work. A beneficial feature in the MLP model used, compared to other ANN architectures such as the RNN and LSTM, is that there is no accumulated error derived from the estimation because MLP does not use the outcome predictions to feed the ANN. For a long time and until now, MLPs have been used effectively in several works to perform accurate predictions [31,32,33]. A multilayer perceptron contains three or more layers that use a nonlinear activation function (usually hyperbolic tangent or logistic function), which allow classifying data that is not linearly separable. Each node in a layer is connected to every node in the following layer, making this kind of ANNs to be fully connected. According to [23], the practical design process should be structured as follows:

- Determine the goals, including what error metric to use, and the expected value for this metric. The problem should drive these goals and error metrics that the application has to solve.
- Establish an end-to-end working pipeline as soon as possible, including the estimation of the appropriate performance metrics.
- Instrument the system adequately to track bottlenecks or issues in the model, such as overfitting, underfitting, or defective data.
- Perform repeatedly incremental changes such as increasing the data set entries, adjusting hyperparameters, or changing algorithms, based on specific findings from the previous instrumentation.

Since the purpose of the work is to create an estimator for roll and sideslip angles in mass production vehicles, the priority is to provide the best forecasts as possible in the least amount of time. As the system is embedded in real vehicles through specific computing units endowed with limited computing capabilities, there must be a balance between the ANN complexity and the prediction accuracy. As error metrics, there has been considered the RMSE (Root-Mean-Square Error) since they allow considering the magnitude of the error to assign a higher loss to larger errors than other metrics (as Mean Absolute Error) do. A large prediction error could result in a dangerous situation, so they need to be minimized.

**Figure 2:**
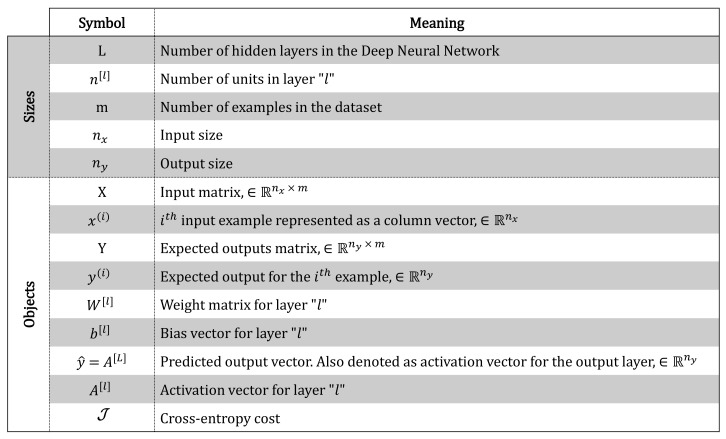
Notation.

The inputs handled to predict the roll ($\phi_{m}$) and sideslip ($\Psi_{m}$) angles (model outcome) are: longitudinal acceleration ($a_{xm}$), lateral acceleration ($a_{ym}$), roll rate (${\dot{\phi }}_{m}$), yaw rate (${\dot{\Psi }}_{m}$), steering angle ($\delta_{v}$), and longitudinal speed ($V_{x}$). These are the typical variables that affect the lateral vehicle dynamics. Notation used in this paper in relation to the DNN model can be seen in Figure 2.

Different factors affect the goodness of a DNN model [23]. The number of data for training and testing the model, the hyperparameters (i.e., learning rate or the number of training iterations) configuration, the number of hidden layers, and the number of units per layer. The original DNN was coded from scratch in Python 3.7 [34] due to the robust set of existing libraries oriented towards matrix processing (NumPy [35]), data processing (pandas [36]) and resulted plotting (matplotlib [37]), plus the ability to run the code in different platforms (from development desktop computers to production embedded devices). The “from scratch” approach was taken to optimize and instrument the code as much as needed. However, to train and test the designed DNN architecture efficiently, the experimental version that led to a proper set of hyperparameters and neuron weights was implemented using Keras [38]. This framework was developed with a focus on enabling fast experimentation (transform the idea into a result with the least possible delay).

**Figure 3:**
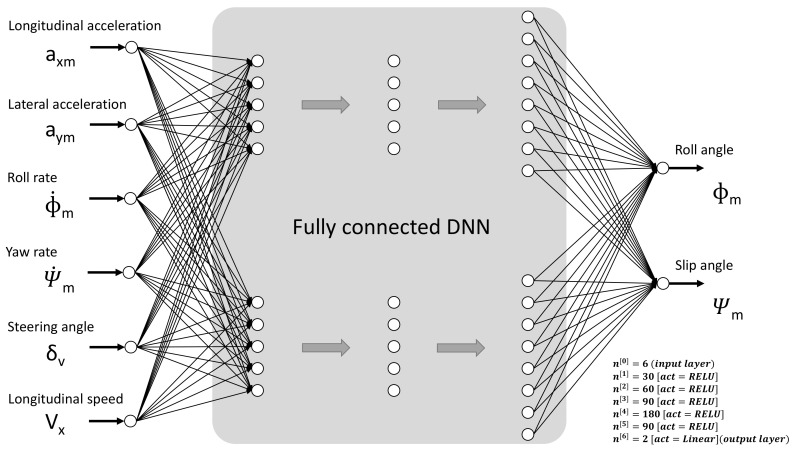
Deep Neural Network configuration used.

Several set-ups related to both parameters and hyperparameters were defined and tested. The best configuration found is represented in Figure 3. This DNN is composed of five hidden layers, six inputs, and two outputs. The network presents thirty units in the first layer, sixty in the second layer, ninety in the third layer, one hundred and eighty in the fourth layer, and finally ninety in the fifth layer. This network configuration was the result of using the conventional method of trial and error. The network was trained using different hyperparameters. Some techniques, as “early stopping” by using a relatively small number of iterations to prevent overfitting, were tested until getting a proper set of hyperparameters. First layer and subsequent ones calculate a vectorized linear function (see Equation (1)) followed by a vectorized activation. The activation function, π(·), for these layers is a RELU (Rectified Linear Unit—see Equation (2)), and the output layer outcome (see Equation (3)) is the result of the linear function, providing a linear regression, suitable for predicting the expected values.

(1) $$A^{\left[l\right]}=\pi \left(Z^{\left[l\right]}\right)=\pi \left(W^{\left[l\right]}A^{\left[l-1\right]}+b^{\left[l\right]}\right)$$

(2) $$RELU\left(Z^{\left[l\right]}\right)=max\left(0,Z^{\left[l\right]}\right)$$

(3) $$\hat{y}=A^{\left[L\right]}=Z^{\left[L\right]}=W^{\left[L\right]}A^{\left[L-1\right]}+b^{\left[L\right]}$$

Once all the layers perform their computations, it is necessary to compute the cost in order to check if the model is learning correctly. The cross-entropy cost J (see Equation (4)) was used for this purpose in order to compare the predicted vs. the expected values:

(4) $$J=-\frac{1}{m}\sum_{i=1}^{m}\left(y^{\left(i\right)}log\left(a^{\left[L](i\right)}\right)+\left(1-y^{\left(i\right)}\right)log\left(1-a^{\left[L](i\right)}\right)\right)$$

After computing the cost, a linear backward (backpropagation) has to be carried in order to update network parameters. To do so, it is required to calculate the gradients: $dW^{\left[l\right]}$ (Equation (5)), $db^{\left[l\right]}$ (Equation (6)) and $dA^{\left[l-1\right]}$ (Equation (7)), respectively:

(5) $$dW^{\left[l\right]}=\frac{\partial J}{\partial W^{\left[l\right]}}=\frac{1}{m}dZ^{\left[l\right]}A^{[l-1]T}$$

(6) $$db^{\left[l\right]}=\frac{\partial J}{\partial b^{\left[l\right]}}=\frac{1}{m}\sum_{i=1}^{m}dZ^{\left[l](i\right)}$$

(7) $$dA^{\left[l-1\right]}=\frac{\partial J}{\partial A^{\left[l-1\right]}}=W^{[l]T}dZ^{\left[l\right]}$$

The linear-activation backward function will be then computed by using the next Equation (8), being π(·) the activation function for RELU and $\pi^{\prime }\left(\cdot \right)$ its derivative:

(8) $$dZ^{\left[l\right]}=dA^{\left[l\right]}*\pi^{\prime }\left(Z^{\left[l\right]}\right)$$

For the last layer, the MSE derivative between predicted and expected normalized values is computed and backpropagated as the first gradient in such a process.

**Figure 4:**
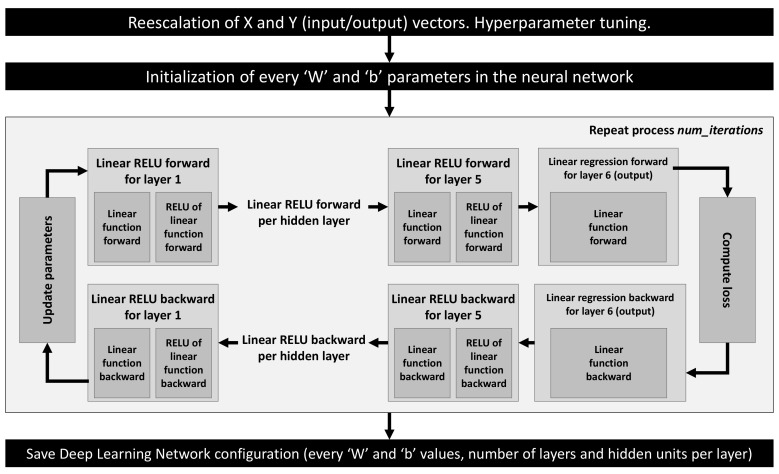
DNN configuration and training procedure followed.

In the last step, when reaching the input layer of the model, it is necessary to update the parameters using gradient descent on every $W^{\left[l\right]}$ and $b^{\left[l\right]}$ for l=1,2,…,L. The equations to do so are:

(9) $$W^{\left[l\right]}=W^{\left[l\right]}-\alpha dW^{\left[l\right]}$$

(10) $$b^{\left[l\right]}=b^{\left[l\right]}-\alpha db^{\left[l\right]}$$

where α is the learning rate hyperparameter. The whole process is repeated for the specific number of iterations defined as a hyperparameter. A graphical description of this process can be seen in Figure 4.

**Figure 5:**
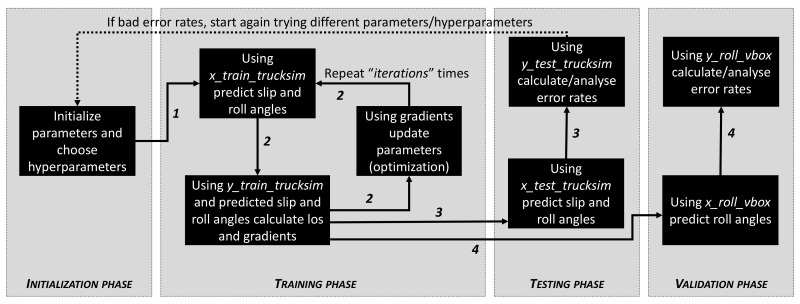
Deep learning approach followed.

A general overview of the approach followed to train and validate the network is presented in Figure 5. The training process is fulfilled with a significant subset of entries from Trucksim^®^ outcome data. Then the testing process is checked against the remaining subset of entries. All the input data are normalized per component. After testing the trained model, it is required to check the predicted error rates, and in case they are not appropriate, new training has to be done after adjusting the hyperparameters (i.e., to prevent overfitting). Once the predicted error rates are adequate, the validation with data acquired from real driving maneuvers can be performed.

#### 2.1.2. Model Dissemination and Extension

The proposed model has been designed as a portable predictor, so the DNN has been implemented in a modular way and using a Python virtual environment, which allows executing the utility function (“predict”) from any computer sharing the same architecture. By trying to minimize the number of external dependencies, and by creating the model from scratch, it is possible to run it from any device supporting a Python interpreter. Besides, the creation of a Docker [39] image is in progress. This image can be deployed in any computing device supporting Docker, and it can also be used not just to provide predictions, but also to continue the extension of the model in the same environment as the one used for its creation. Besides, the simplicity of the communication interface provides easy integration with other systems that may benefit from sideslip and roll angle predictions, as the perception-based knowledge model proposed in [40]. The synergy with those kinds of systems may lead to a complex representation and prediction model for vehicular dynamics at different scales, being suitable to enhance vehicular autonomy, by providing more complex and accurate predictions by fusing multiple sensors and information sources.

## 3. Datasets

**Figure 6:**
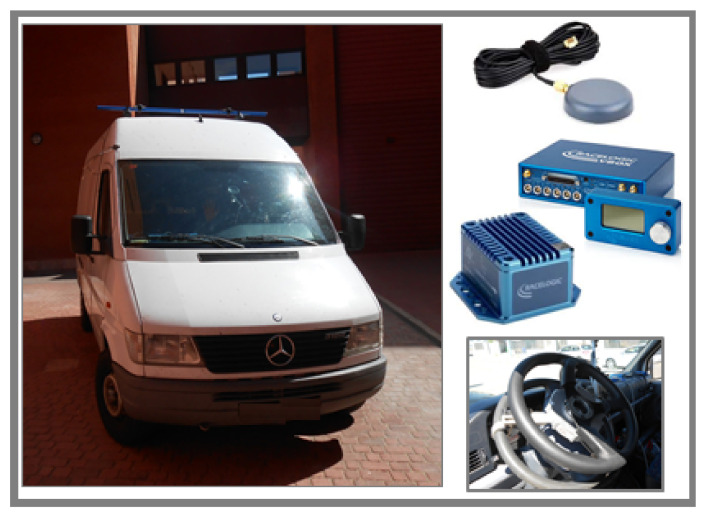
Real Mercedes Sprinter van and instruments setup.

The training dataset has to be adequately chosen to take into account all driving situations and conditions. The training dataset has been obtained from a previous experimentally validated Trucksim^®^ van model employing the real Mercedes Sprinter depicted in Figure 6 [41]. The model parameters were adjusted by trial and error according to the differences between the experimental and simulation data. Trucksim^®^ is a software widely recognized and validated for application in vehicle dynamics. The effectiveness of this software allows us to simulate risky maneuvers that would not be possible, for security reasons, to recreate them in reality. Besides, the convenience of the software allows us to modify the road conditions quickly, allowing a much more comprehensive range of tests when obtaining valid information. Finally, the use of simulation software guarantees the reproducibility of the tests. Trucksim^®^ requires to model the vehicle dynamics before simulating it.

**Figure 7:**
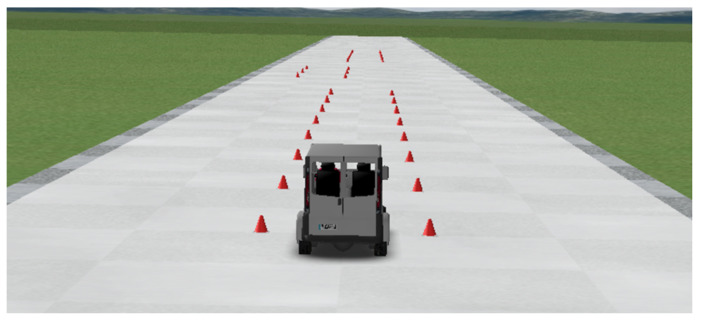
Simulated scenario for a Double Lane Change (DLC) maneuver in Trucksim^®^.

**Figure 8:**
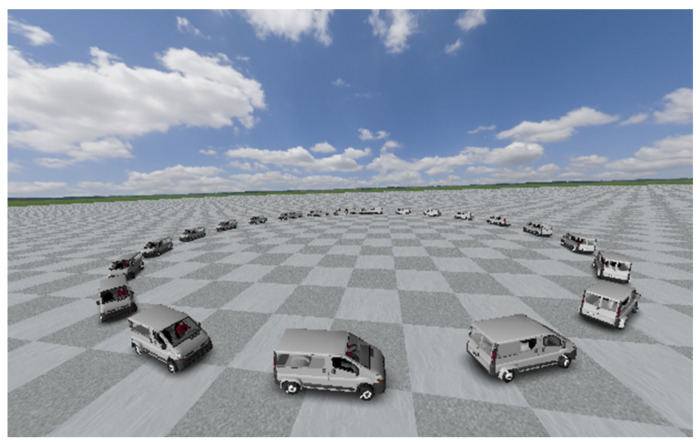
Simulated scenario por a Left J-turn maneuver in Trucksim^®^.

In this work, authors considered employing a van, as this type of vehicle has, on average, a higher center of gravity, which makes them more prone to loose lateral stability. The maneuvers simulated in the software are common ones in the experimentation of vehicle dynamics. Double lane change (DLC), Figure 7, JTurn (JT), both dextro-rotatory and levorotatory, Figure 8, and sine steering maneuvers have been used for the training of the proposed learning-based DNN. Each of the maneuvers has been performed in a range from 20 km/h to 120 km/h. Those tests in which the van virtually did not maintain stability have been discarded since the data is no longer valid for network training. Table 1 shows a summary of the data set used for the training and test of the DNN. More than 400,000 entries were used considering a training-testing ratio of 70:30.

The data from Trucksim^®^ van physic model simulated driving maneuvers are stored in a CSV file (Comma Separated Values) format. Working directly with a CSV file in Python is not as efficient as using other formats such as Pandas DataFrames [36] or NumPy matrices [35], which also support vectorized operations and broadcasting.

A remarkable characteristic of Deep Neural Networks is that given enough data corresponding to training examples—the structured set of inputs and expected outputs; they are accurate at figuring out the functions that accurately map from inputs to expected results. In the specific case of this work, the training examples contained more than 400,000 entries, which led to an efficient and accurate predictor.

The first step taken was to convert the heterogeneous data sets from CSV into a combined and structured data set. HDF5 [42] format was used, as it is high-performance data management and storage suite that can be applied to manage, process, and store heterogeneous data. HDF5 also allows a fast I/O processing that provides fast access times and storage space optimizations, and portable storage within a self-describing file format. Besides, all data and metadata can be passed along in one file (a useful feature to preserve the data set headers, in the same way as in CSV) and without a limit on the number or size of data objects in the collection, giving great flexibility for big data. Even more, it comes as a multi-platform software library that implements a high-level API with interfaces in multiple programming languages such as Python. Before converting CSV files into HDF5 collections, they had to be parsed by a Python script explicitly made for this task. This script is not just cleaning the files discarding not used variables, but also is in charge of shuffling and splitting the Trucksim^®^ data set into training and testing subsets.

There are as many rows as variables to learn from, and as many columns as training examples. Analogously, as many rows as expected values (two, sideslip and roll angles), and as many columns as input training examples. The testing and validation phases use the corresponding data sets stored as collections in the aforementioned HDF5 file. Finally, the trained DNN weights are also stored in a specific HDF5 file produced by Keras, which later can be loaded to perform further predictions.

## 4. Results and Discussion

**Figure 9:**
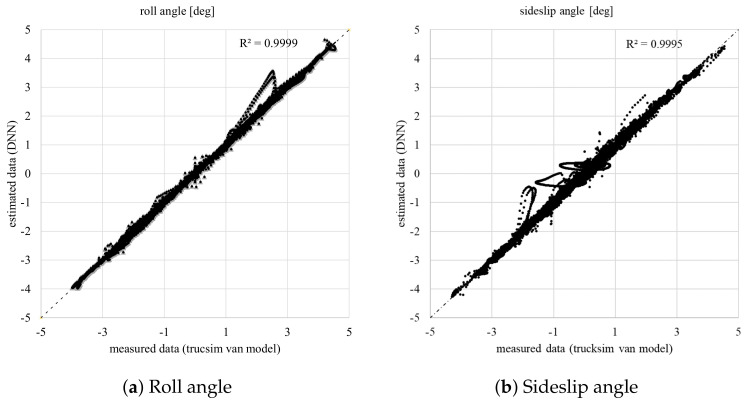
Comparison between measured data from the TruckSim^®^ van model and the estimated data from DNN.

In this section, simulation results from the learned DNN estimator using the training dataset depicted in Table 1, are firstly presented. Secondly, the proposed estimator is experimentally validated using a real van. Figure 9 shows the comparison between the roll and sideslip angles obtained by Trucksim® with those estimated by the proposed DNN for all maneuvers used during the training. The regression plots’ examination reveals that the proposed DNN learns reasonably good for all data sets, with R values close to 0.999.

In addition to the graphical evidence of the effectiveness of the proposed DNN, a quantitative study bears in mind the RMS (Root Mean Square), maximum, and norm errors that have been calculated (Table 2). The norm error is obtained from the following equation [41]:

(11) $$E_{t}=\frac{\varepsilon_{t}}{\sigma_{t}}$$

where

$$\varepsilon_{t}^{2}=\int_{0}^{T}{\left(\lambda_{measured}-\lambda_{estimated}\right)}^{2}dt$$

$$\sigma_{t}^{2}=\int_{0}^{T}{\left(\lambda_{measured}-\mu_{measured}\right)}^{2}dt$$

$\lambda_{measured}$ and $\lambda_{estimated}$ are the measured and estimated roll and sideslip angles, respectively, and $\mu_{measured}$ is the mean value of the roll and sideslip angles obtained from Trucksim^®^ during the period T.

**Figure 10:**
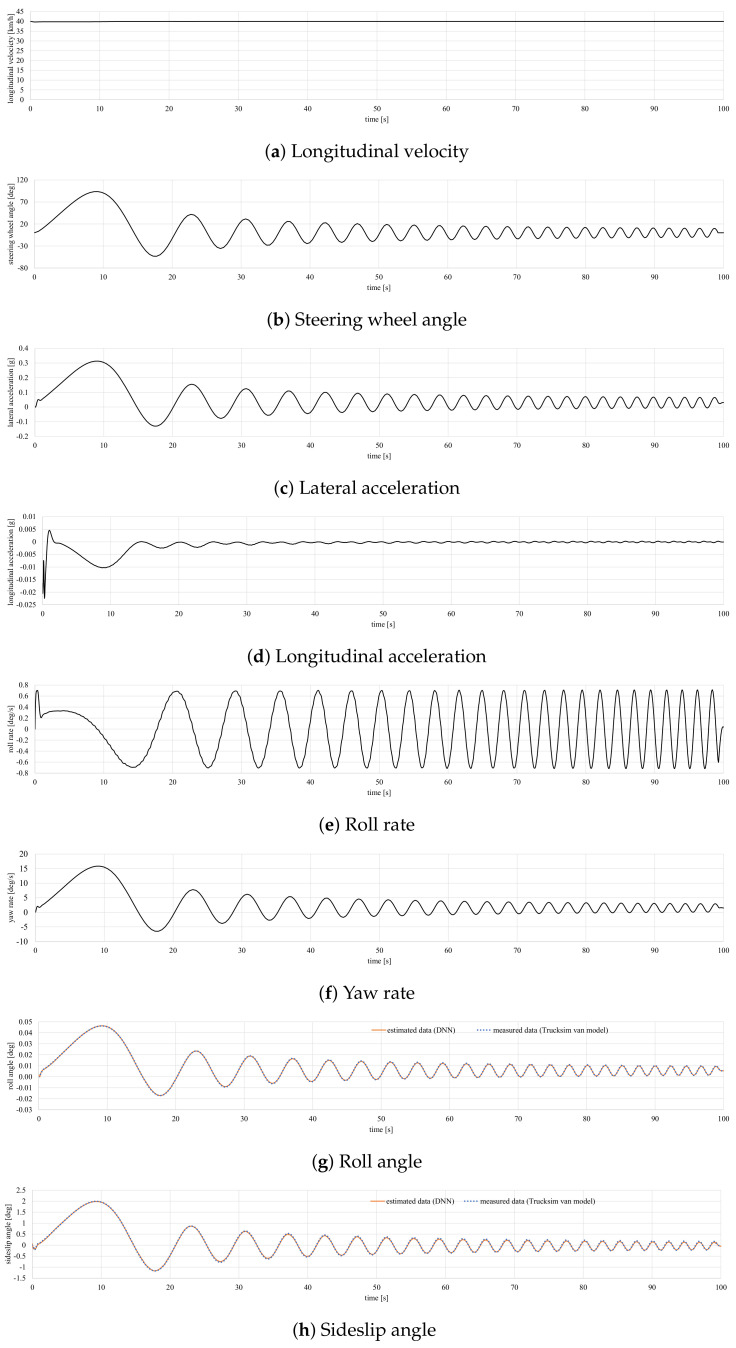
Simulation results for the validation test.

To corroborate the proposed estimator’s good performance, a new dataset has been selected for validation. This dataset consists of a handling sine sweep maneuver with a steering wheel angle ranging from $\pm 90^{{}^{\circ}}$ to $\pm 10^{{}^{\circ}}$ and a frequency from 0.5 to 0.2 Hz with a friction coefficient of 0.85. The vehicle moves at a velocity of 40 km/h. The simulation results are depicted in Figure 10. Table 3 shows the RMS, maximum and norm errors. Results show that the proposed DNN model performs quite well.

Once the proposed estimator has been trained and validated from Trucksim^®^ van model, the real Mercedes Sprinter van (see Figure 6) has been used for its experimental validation. The studied test-bed van is composed of different experimental kits:

1. Vbox 3i dual antenna data logger from Racelogic.
2. An IMU (Inertial Measurement Unit) sensor from Racelogic mounted near the center of gravity (COG) of the vehicle to provide measurements of the roll and yaw rates and longitudinal and lateral accelerations.
3. Two GPS antennas from Racelogic to provide measurements of the roll and sideslip angles (Ground Truth). The dual antennas must be positioned transverse to the direction of movement to precisely determine the roll angle.
4. Steering angle sensor MSW 250 Nm from Kistler.

**Figure 11:**
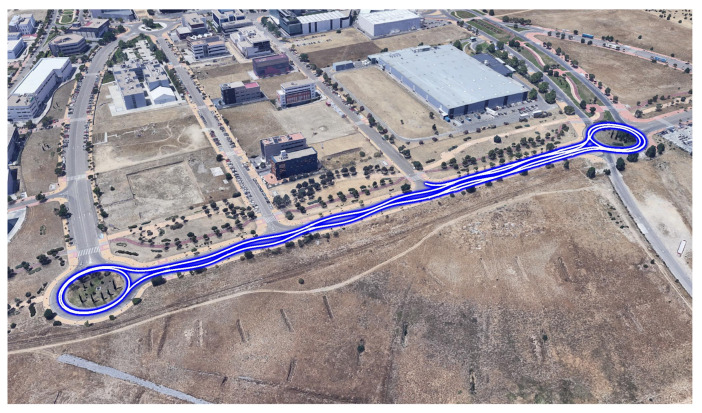
Experimental scenario for the validation test.

**Figure 12:**
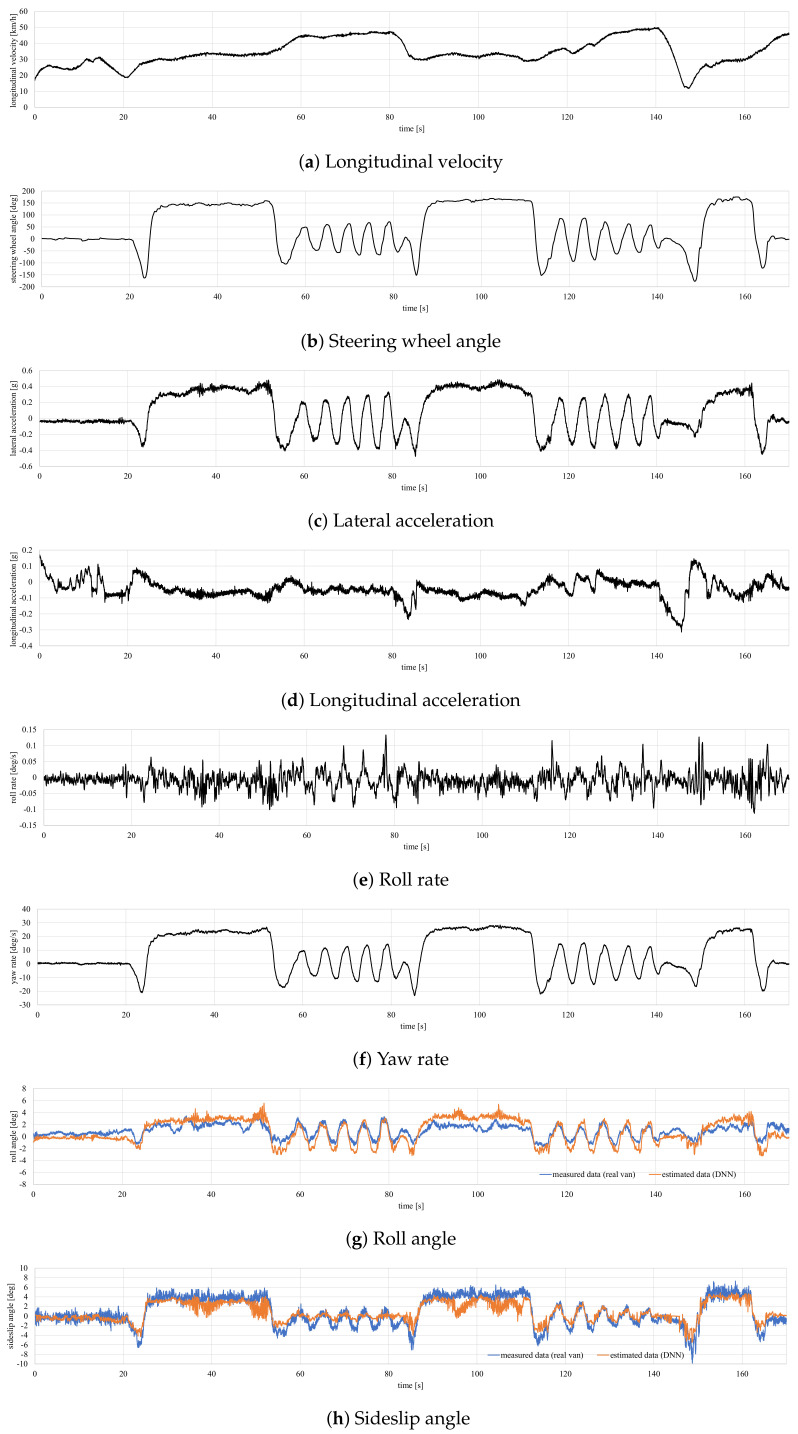
Experimental results for the validation test.

The experimental scenario and the real maneuvers performed by the van are depicted in Figure 11. The van’s sequence of maneuvers is a straight line during the first 20 s, J-Turn maneuvers in the roundabouts and slalom maneuvers on the straight sections of the road. The van speed profile is shown in Figure 12.

The results are depicted in Figure 12. Table 3 shows RMS, maximum, and norm errors for both roll and sideslip angle predictors using the validation data set from real maneuvers logged with a Racelogic VBOX IMU. As can be seen, the trained DNN behaves slightly better, estimating the roll angles than the sideslip angles. For a better estimation, different filters such as Kalman filter [17,41] and $H_{\infty }$ [18] can be used as it has been done in previous works, to filter noise and minimize the errors’ estimation. One of the primary sources of error in the proposed DNN is that road irregularities and road banks have not been considered. Although these disturbances have not been taken into account, the obtained errors in the estimation of the roll and sideslip angles are deemed acceptable. These disruptions could be estimated by designing more complex observers [43,44]. Nevertheless, this involves installing more sensors on the vehicle, increasing its cost, and the overall computing time.

Finally, a comparison with other methods previously proposed for the estimation of roll and sideslip angles has been made (see Table 4). These methods do not estimate both angles jointly in contrast to the proposed method. Considering the roll angle estimation, the van used was the same as in Boada et al. [18]. As is depicted in Table 4, the proposed method achieves better results both in maximum and norm errors. On the other hand, if the sideslip angle is taking into account, the method proposed shows better simulation results than the method proposed by Kim et al. [27] as is reported in Table 3. Regarding experimental results, although the RMS and maximum errors obtained in [27] are smaller, it should be noted that the type of vehicle used to carry out the essays is different. The van used in this work is characterized by a high CoG (Center of Gravity), and it is equipped with a soft suspension which involves a more significant chassis movement, thereby affecting the longitudinal and lateral load transfer and the vehicle’s dynamic.

Considering the size of training data (more than 400 K elements), and the DNN width and depth, each training block took initially around 40 min. After including Keras in the training code, this time was reduced to one fourth. The implemented DNN uses vectorization and efficient matrix handling libraries, such as NumPy, which made this process faster. With other configurations, including more profound and broader networks, the times varied, requiring initial and sometimes more than seven hours per training process. Regarding the predictions, in the same type of computer, processing 70 K elements take around 3 s. This model has to be tested in small embedded systems, such as the Raspberry Pi 4 Model B+ prototyping boards, to assess that predictions are provided at least at a sustained rate of 50 Hz (20 ms). This real-time constraint must be satisfied with safety systems. The performance results from different studies, where the authors used the previous Raspberry Pi 3 Model B+ to run deep learning models [45,46,47] in Keras and TensorFlow, and all of them heavier than the one presented in this work, show that the average time for evaluating an input is ranged from 0.001 s to 0.12 s. Given that the projected hardware to be used is the Raspberry Pi 4 Model B+, which has been proved to perform more than four times better than the former model [48], it seems feasible that the system will satisfy the real-time constraint mentioned before.

## 5. Conclusions

The main objective behind this work was to define a proper Deep Learning approach to simultaneously estimate sideslip and roll angles in commercial vehicles by capturing the required input values via a set of sensors such as IMU and steering angle sensor. Specifically: longitudinal acceleration, lateral acceleration, roll rate, yaw rate, and the steering angle. The DNN configuration was designed trying to minimize the impact in processing the outcomes; this is, looking for a compromise between width, depth, and prediction quality after considering the hardware restrictions and limited performance of vehicular embedded systems. Besides, the proposed DNN does not use previous information from the sensors but only the one that is provided at that step of time, so that there are no stability problems associated with an accumulated error. Several configurations were trained and tested, altering both the network shape and the hyperparameters set. The validation was conducted in two phases, the first one, using a subset of the outcome data from Trucksim^®^, a well known vehicular simulator, that was used to model the physics of a real vehicle (van), and to use that van in a series of simulations consisting of performing different driving maneuvers. In the second validation phase, and after passing the quality check by analyzing the predictor errors against the test data subset, the model was assessed against the data captured by a ground truth device, specifically a Racelogic VBOX IMU, used in real driving maneuvers with a similar van such as the one modeled in Trucksim^®^. Finally, errors against this real data were also analyzed to check if the predictor is suitable for being used in real vehicles, which seems to be the case.

### Future Work

Considering the outstanding predictions returned by the implemented DNN model, it is planned to integrate it into an enhanced version of the IoT architecture proposed in previous works. It is composed of a Raspberry Pi 4 Model B+ (4 GB), a BNO55 IMU Shield, the Racelogic VBOX used in this study, a laptop, and a 4G mobile hotspot device to provide connectivity among the prototyping board (RPi4) device and the laptop. At the same time, this IoT ecosystem will be integrated into two different testing vehicles (the Mercedes Sprinter van used to capture the validation data in this work, and a buggy). The objective of this new experiment is to validate both the roll and sideslip angles predicted in real-time, comparing the results with the ground truth device (Racelogic VBOX). On the other hand, there are also research efforts put on improving the values of the hyperparameters, and the DNN structure/combination of activation functions in the inner hidden layers, trying to increase even more the outcome precision and reducing the computational cost to make it perfectly suitable for its integration into ultra-low-cost embedded systems.

## Figures and Tables

**Table 1:** Training DNN data sets (Trucksim^®^).

Road Friction Coefficient	Maneuver	Speed (km/h)	Steering Angle (deg)
0.3	Left DLC	20, 30, 40, 50, 60, 70	-
0.5	Left DLC	20, 30, 40, 50, 60, 70, 80, 90, 100, 110, 120	-
1	Left DLC	20, 30, 50, 60, 70, 80, 90, 100, 110, 120	-
0.3	Right DLC	20, 30, 40, 50, 60, 70	
0.5	Right DLC	20, 30, 40, 50, 60, 70, 80, 90, 100, 110, 120	-
1	Right DLC	20, 30, 40, 50, 60, 70, 80, 90, 100, 110, 120	-
0.3	Left J-Turn	20, 30, 40, 50, 60, 70, 80, 90, 100, 110	40, 60, 90, 100, 120
0.5	and		
1	Right J-turn		
0.5, 0.85	Sine steering	30 to 60 km/h in 30 s	±60 (0.2 Hz, 0.5 Hz)
0.5, 0.85	Sine steering	30 to 60 km/h in 30 s	±90 (0.2 Hz, 0.5 Hz)

**Table 2:** Error measurements for the training and test dataset.

	Trucksim^®^ Van Model	
	Roll Angle	Slip Angle
**RMSE [°]**	0.018	0.033
**E*_max_* [°]**	1.05	1.39
**E*_t_* [-]**	8.11 × 10^−5^	3.83 × 10^−4^

**Table 3:** Error measurements for validation datasets.

	Trucksim^®^ Van Model		Real Van	
	Roll Angle	Slip Angle	Roll Angle	Slip Angle
**RMSE [°]**	0.018	0.024	1.19	1.40
**E*_max_* [°]**	0.11	0.094	3.5	6.36
**E*_t_* [-]**	0.017	0.040	1.14	0.48

**Table 4:** Comparison with other methods for roll and sideslip angles estimation.

		Roll Angle	Sideslip Angle	
		Boada et al. [18]	Kim et al. [27]	
Type of Test		Experimental	Simulation	Experimental
**Errors**	**RMSE [°]**	-	0.14–0.96	0.12–0.19
	**E*_max_* [°]**	5.5	0.92–3.84	0.75–1.92
	**E*_t_* [-]**	1.95	-	-
**Type of vehicle**		van	sedan	
**Type of NN**		MLP	LSTM + deep ensemble	

## Abbreviations

The following abbreviations are used in this manuscript:

ABS	Anti-Blocking System
ANN	Artificial Neural Network
CoG	Center of Gravity
DNN	Deep Neural Network
ESC	Electronic Stability Controllers
GPS	Global Positioning System
IMU	Inertial Measurement Unit
LSTM	Long Short-Term Memory
MLP	Multilayer Perceptron
MSE	Mean Squared Error
NLP	Natural Language Processing
RMSE	Root-Mean-Square Error
RNN	Recurrent Neural Networks
